# Peer Learning Has Double Effects in Clinical Research Education: A Qualitative Study

**DOI:** 10.1155/2024/5513079

**Published:** 2024-01-27

**Authors:** Hiro Nakao, Osamu Nomura, Chie Nagata, Akira Ishiguro

**Affiliations:** ^1^Center for Postgraduate Education and Training, National Center for Child Health and Development, 2-10-1 Okura, Setagaya-ku, Tokyo, Japan; ^2^Department of General Pediatrics & Interdisciplinary Medicine, National Center for Child Health and Development, 2-10-1 Okura, Setagaya-ku, Tokyo, Japan; ^3^Department of Health Sciences Education, Hirosaki University, 5 Zaifu, Hirosaki, Aomori, Japan

## Abstract

**Background:**

Peer learning has been recognized for its effectiveness in health professional education. However, its effects on clinical research education are not clear and were explored qualitatively in this study.

**Methods:**

The peer-learning method was implemented in a clinical research education seminar for early-career physicians at a children's and mothers' hospital in 2019. We conducted semistructured interviews with participants about peer-learning experience and qualitatively analyzed verbatim transcripts using Engeström's “activity theory” framework.

**Results:**

From framework analysis, learning processes were extracted mainly in four domains, namely, (a) instrument and its usage: research design and its match with research question, (b) outcome: research result, (c) community: seminar, and (d) division of labor: roles of participants and staff.

**Conclusions:**

In this report of a peer-learning trial in postgraduate clinical research education, the following two pathways of peer-learning effects were abstracted. The indirect pathway was the presentations by experienced participants providing concrete examples of research processes. The direct pathway was the questions from experienced participants to beginners about specific and concrete questions. There were also two points to consider in peer learning in clinical research education: gaps in premise knowledge and beginners' frustration about expected outcomes. We believe that these extracted pathways and points imply the significance and considerations for continuing the peer-learning trial in clinical research education. Future tasks are to promote clinical research education with a view to the learning effects, not only on individuals, but also on groups.

## 1. Introduction

### 1.1. Background

Peer learning is an educational method in which colleagues teach each other, and its effectiveness has been recognized in the education of health professionals and medical students [1–7]. In particular, peer learning has been reported to be effective in the education of medical students, both for those who teach and those who are taught [1, 2]. Recently, it was reported that peer learning was effective in reducing stress and maintaining the motivation of medical students during the COVID-19 crisis, in terms of psychological contact, cooperation, communication, improvement, and sharing of ideas and feelings [3]. Though most reports of peer learning in postgraduate healthcare education were from nurses and radiologists, the few articles on peer learning in education for foundation doctors similarly reported benefits to both the teacher and those being taught and the importance of peer feedback [6, 7].

With the benefits of peer learning, we considered applying this type of educational model to clinical research education. As evidence-based medicine has been taking a very central position in clinical medicine, there have been increasing reports of educational programs for clinical research literacy and skills [8–11] using methods other than peer learning. The effects of using the peer-learning method in clinical research education are not yet clear. In addition, research education is becoming increasingly important in the training of pediatrics residents in Japan [12, 13]. With approximately 40 residents training in the National Center for Child Health and Development (NCCHD) every year [14], research education for beginners is as important as skill sophistication for research experts concerned with evidence generation at NCCHD because it is important to train more research mentors who can provide research opportunities that enhance residents' scholarly activities [15]. The aim of the current study was to explore the effects of peer learning on beginners' research competency in clinical research education at a children's and mothers' hospital.

### 1.2. Research Question and Theoretical Framework

We trialed peer learning in clinical research education as part of the postgraduate training of physicians at NCCHD. We employed a near-peer learning method among the various peer-learning methods, in which the distance between those who teach and those being taught, the learning group's size, and the learning environment's formality are all high [5]. In any case, we were concerned that peer learning might impose some burdens on beginner learners such as prior preparation, extra time outside of lectures, or the stress of responding flexibly to questions and opinions on the spot. Therefore, we conducted a pilot study with the research question of how peer learning in clinical research education was experienced by the beginner learners themselves, to help us decide whether there were positive aspects of peer learning that could outweigh the burden on the beginner learners.

For this research question, we adopted Engeström's activity theory as our theoretical framework [16]. The activity theory model consists of two triangles, small and large. The small triangle means an individual activity, in which a “subject” acts on an “object” using an “instrument” to produce an “outcome,” while the large triangle, which contains the small triangle, is a “community” which collectively cooperates in activities through “rule” and “division of labor” toward the “outcome.” Activity theory is often referred to in the practice and analysis of cooperative learning in the community, such as in active learning in and out of class and on-the-job learning at workplaces [17–24]. In recent years, applications in the healthcare field have also been attempted [21–24]. As for peer learning, Vygotsky's educational psychology is also referred to as one of the theoretical foundations [5, 25]. Activity theory possesses an aspect of extending Vygotsky's theory of individual learning to the group level [16]. In addition, activity theory was reported to be applied to the analysis of peer learning in clinical medical education [26]. Therefore, we hoped that activity theory would provide suggestions for analysis and practice in our peer-learning trial on postgraduate education.

## 2. Methods

### 2.1. Seminar

NCCHD is a children's and mothers' hospital in Japan with approximately 400 pediatric and 100 maternity beds. The Practical Clinical Research Advanced Course (“seminar” for short) at NCCHD was held as a part of postgraduate training for early-career physicians once every two weeks from June to December 2019, for a total of 12 sessions. The seminar was delivered for approximately two hours per session with the aim of teaching the fundamentals of clinical research to further develop research in child healthcare. The seminar instructors were staff with extensive experience and skills in clinical research. Recruited participants were mainly early-career clinical physicians working at NCCHD who were willing to conduct clinical research. The actual participants were 12 early-career physicians: one from obstetrics, two from neonatology, three from pediatrics, three from anesthesiology, and three from surgical departments. Eight of them were “experienced” who had attended the course the previous year, had started clinical research, and were recruited to reflect on their learning and play important roles in peer learning as noted below. The remaining four were “beginners” attending the course for the first time that year.

The teaching method of the seminar was composed of lectures and peer learning. Lectures were given on clinical research methods and design, research ethics, research protocols, sampling, measurement, analysis, and grant application for about one hour of each two-hour seminar by one instructor per lecture. The remaining hour was devoted to peer learning, consisting of presentations and discussions among participants on clinical questions (CQ), research questions (RQ), and research plans of their own. Each peer-learning session was attended by all participants and four to six instructors who facilitated discussions. In order to structure peer learning, we recruited the previous year's participants as “experienced” peers, resulting in eight “experienced” participants as noted above.

### 2.2. Data Collection

This pilot study employed a purposeful sampling method of the beginners in the course. Three out of four beginners consented to a 10-minute-long semistructured interview about peer learning. One experienced participant was interviewed for data triangulation of interview information. The required questions in the interview guide were on their experience and what they noticed in the peer-learning session, and open-ended questions about their frank feelings about peer learning. The interviews were recorded and transcribed verbatim. All personal information, including names, affiliations, and research themes, were removed from the verbatim transcripts.

### 2.3. Analysis

The anonymized verbatim text was analyzed using framework analysis [27, 28], a qualitative method of analysis. Framework analysis uses structured concepts, which are induced from data and deduced from theory, as a framework to describe and abstract qualitative data. Framework analysis proceeds through the following steps: data familiarization, framework application, indexing, charting, and interpretation. First, the familiarization step provides the researcher with an initial understanding of the data, topic extraction, and induction. We supplementally employed text mining with KH Coder [29, 30] (version 3) in this data familiarization step in order to capture the trend of topics preliminary to coding, as one researcher was not involved in the seminar or interview. The next framework application step combines induction from data and deduction from the theoretical framework into structured concepts as a framework. As a theoretical framework, we used Engestrӧm's “activity theory” [16], which is often referred to in non-self-taught, group learning [17–24] as noted earlier, and the theory decides the research paradigm as constructivism [31]. The researcher then systematically applies the conceptual framework [32] to the intercepted and decontextualized data in the indexing step and recontextualizes and processes the indexed data in the charting step. Finally, the researcher summarizes and interprets the charted data according to the research question in the interpreting step.

This study was carried out by four researchers consisting of three staff members and one not involved in the seminar for investigator triangulation. One researcher, who was one of the facilitators of peer learning, consistently performed the interviews, and two researchers, including one seasoned in qualitative research, mainly analyzed the data and all checked the analysis. This paper was written according to the Standards for Reporting Qualitative Research [33].

### 2.4. Ethics

Written consent was obtained for the interviews, recording, and publication of the study. The study was approved by the Ethics Review Board of NCCHD (Review No. 2211).

## 3. Results

### 3.1. Text Mining

**Figure 1:**
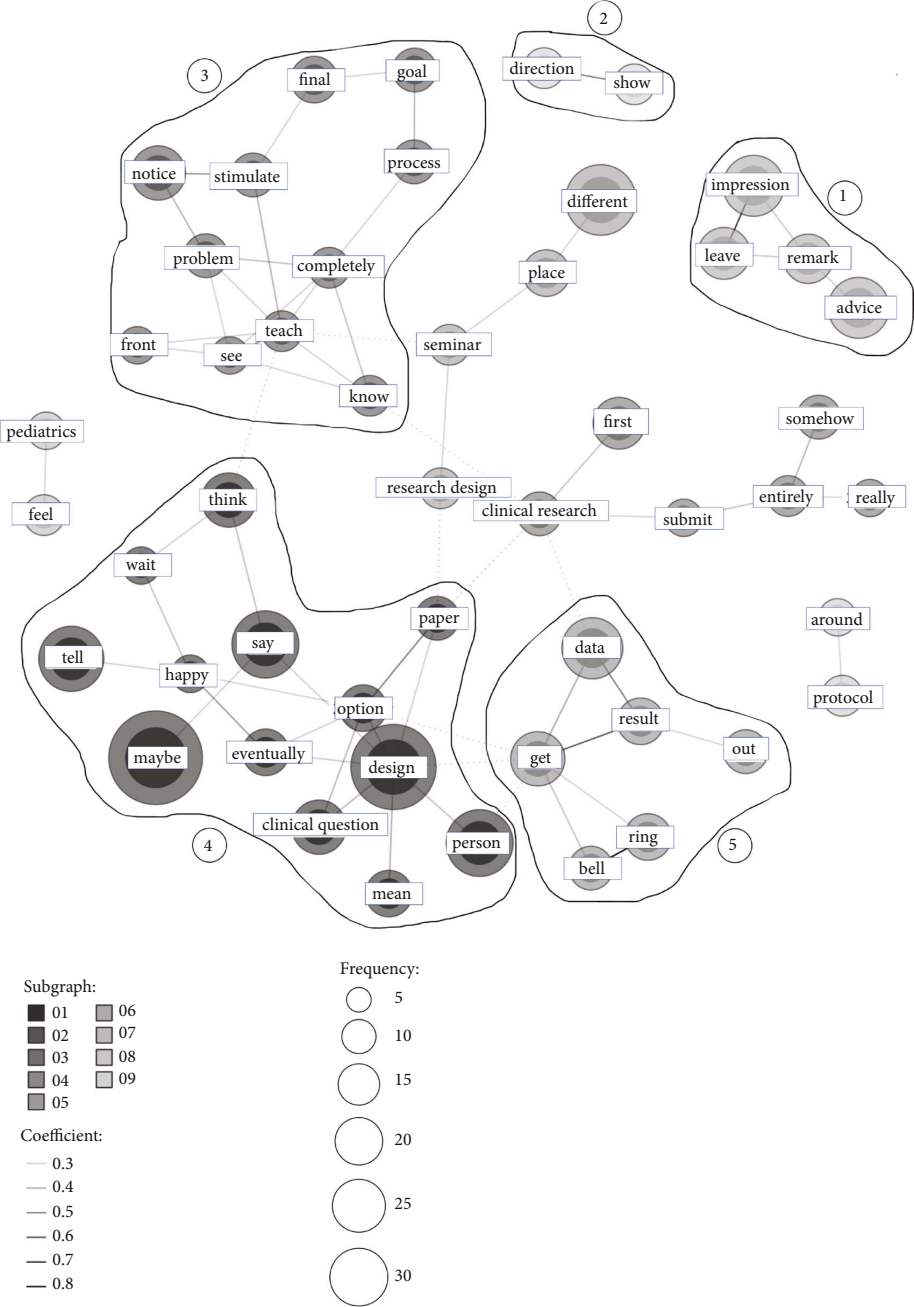
Cooccurrence network [29] written by the KH coder. The topics of the interview were (1) about “remarks” and “advice” that “leave” an “impression”; (2) about “showing” a “direction”; (3) about “teaching” and “stimulating” people to “notice”, “see”, and “know” the “problems” or “process” leading to the “final” “goal”; (4) about “telling” and “saying” to “think” about “options” of “designs” for “clinical questions” in the “paper”; and (5) about “ringing” a “bell” about “getting” “data” and “results” being “out.” There also were words difficult to capture as clumps in the network diagram. These extracted topics and text examples are listed in Table 1.

Figure 1 shows the cooccurrence network [29] made by the KH coder. In this first familiarization step, we included not only the participants' answers but also the interviewer's questions in the analysis to fully understand the context. The researchers compared this network diagram with the raw interview texts, again and again, to capture topics and achieve familiarization with the data. The representative topics extracted in this step were the following: (1) about “remarks” and “advice” that “leave” an “impression”; (2) about “showing” a “direction”; (3) about “teaching” and “stimulating” people to “notice,” “see,” and “know” the “problems” or “process” leading to the “final” “goal”; (4) about “telling” and “saying” to “think” about “options” of “designs” for “clinical questions” in the “paper”; and (5) about “ringing” a “bell” about “getting” “data” and “results” being “out.” Words that were difficult to capture as clumps in the network diagram were also reviewed and carefully read for the context of occurrence in the raw texts.  Table 1 lists these extracted topics and text examples.

### 3.2. Label Generation

**Figure 2:**
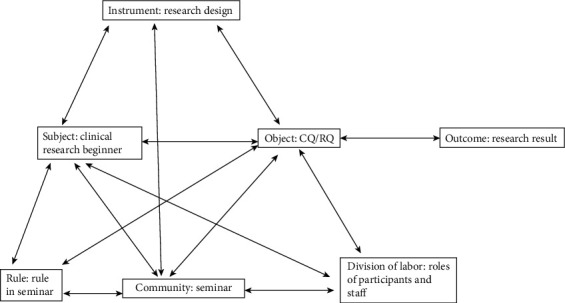
Label generation in the clinical research peer learning using the activity theory [16] framework by Engestrӧm. In the context of the peer learning in the current clinical research seminar situation, the small triangle of activity theory was supposed to correspond to individual research activity and the large triangle to the peer-learning scene in the seminar. Therefore, each activity theory framework category and each corresponding label (separated by a colon) were generated as follows: outcome: research result; subject: clinical research beginner; object: clinical question (CQ)/research question (RQ); instrument: research design; community: seminar; rule: rule in seminar; division of labor: roles of participants and staff. This conceptual framework meant that each clinical research beginner (subject) carried out clinical research activities on CQ/RQ (object) using research designs (instrument) to bear research results (outcome) and the seminar (community) collaborated in these clinical research activities under rules in seminar (rule) and each role of participant and staff (division of labor). CQ: clinical question; RQ: research question.

After data familiarization in Figure 1 and Table 1 above, we generated labels as in Figure 2 by using the activity theory model by Engestrӧm [16]. This labeling corresponds to the framework application step of analysis [27, 28], which combines the induction from the familiarized data and the deduction from the theoretical framework to generate a conceptual framework [32] in the specific context of the current study. In the context of the peer learning in the current clinical research seminar situation, the small triangle of activity theory was supposed to correspond to individual research activity and the large triangle to the peer-learning scene in the seminar. Therefore, each category in the activity theory model and each corresponding label (separated by a colon) was generated as follows: outcome: research result; subject: clinical research beginner; object: clinical question (CQ)/research question (RQ); instrument: research design; community: seminar; rule: rule in seminar; division of labor: roles of participants and staff. This conceptual framework meant that each clinical research beginner (subject) carried out clinical research activities on CQ/RQ (object) using research designs (instrument) to bear research results (outcome) and the seminar (community) collaborated in these clinical research activities under rules in seminar (rule) and each role of participant and staff (division of labor).

### 3.3. Process Extraction

Table 2 shows the primary results of the indexing and charting steps [27, 28]. In these steps, interview texts were intercepted, decontextualized, and coded using the labels generated in Figure 2. Then, the coded texts were recontextualized and process-formed. The analysis concentrated on the interviewee's narratives in these central steps.

Outlined below are extracted processes and text excerpts for each label used.

- Instrument: research design

Participants were aware that difficult designs can be learned when encountering the object (CQ/RQ) requiring that design.

“*If I experienced the same kind of difficult design for my own research, I would probably be able to understand.*”

Participants also noted it is important to know there is a wide variety of research designs in the clinical research world.

“*If you have a clinical question, there are a number of things you can do.*”

Participants remarked on the importance and difficulty of learning the usage of the instruments, that is, match of research design with CQ/RQ and the significance of experiences to master the match.

“*The most difficult part is to decide what kind of research design to use. I had a hard time in this area, so I have the experience of overcoming this difficulty.*”

(b) Outcome: research result

Participants indicated it is helpful to reexamine designs by looking backward from the expected results that can be obtained by that design.

“*Since you are interested in this, would you be happy if you could get this kind of data from that design, for example?*”

Participants, particularly beginners, expressed frustration with the timeline of the required outcomes.

“*I feel it's necessary to move fast toward that goal.*”

(c) Community: seminar

Participants recognized moments of silence in the seminar when they could not continue discussions because of the gap in the participants' background knowledge of the research designs.

“*Maybe it's because something like a research framework isn't formed for beginners and ‘experienced' participants have experience and a framework.*”

Participants also found it significant and interesting to make and hear presentations beyond one's specialty.

“*They're talking about areas that are completely unfamiliar to me, outside of my area of expertise, and I think it's fun to hear about other areas.*”

(d) Division of labor: roles of participants and staff

Participants pointed out different roles between staff and “experienced” participants.

“*I think ‘experienced' participants are going to encourage, or rather stimulate, or interact with beginners and get the discussion going. Finally, staff will put a sort of seal on it.*”

Participants perceived two important roles of “experienced” members. The first role was their presentation providing concrete examples of CQ/RQ, designs, and process to outcomes.

“*In their presentation, I was very glad to see the precedent of how ‘experienced' participants have done this kind of process and had these problems.*”

The second was specific and concrete questions from “experienced” learners based on recent research experiences of their own.

“*I think that was probably the point they were struggling with so much.*”

## 4. Discussion

### 4.1. Analysis Summarization

Effects of peer learning trialed in a clinical research education seminar for physicians in a children's and mother's hospital were explored through a framework analysis using Engestrӧm's “activity theory” on the verbatim transcripts of semistructured interviews with the participants.

**Figure 3:**
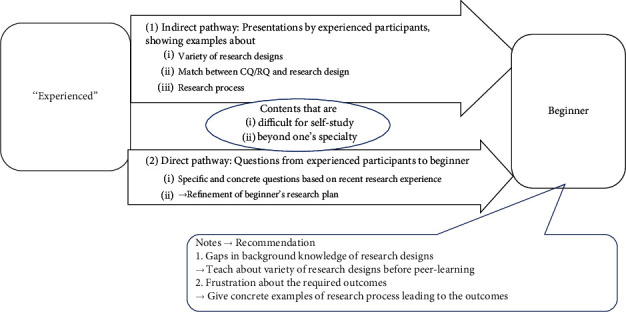
Effects of peer learning in clinical research education and notes to consider. From the charting of the qualitative analysis in Table 1, we abstracted indirect and direct pathways of peer-learning effects on clinical research education. The indirect pathway was the presentations by “experienced” participants, showing concrete examples about a variety of research designs, match between CQ/RQ and designs, or research processes. The direct pathway was the questions asked by “experienced” participants to beginners, which were specific and concrete based on their recent research experience and helped beginners refine their research plans. Both pathways seemingly reached contents difficult for self-study or beyond one's specialty. It appeared to be noted that there were gaps in background knowledge among participants and that some beginners were frustrated by the required results. CQ, clinical question; RQ, research question.

Based on the above charting result in Table 2, we considered that peer-learning effects on beginners in clinical research education, which was the purpose of this study, could be summarized in two main ways, indirect and direct, as shown in Figure 3. This summarization corresponds to the interpreting step of framework analysis [27, 28].

The indirect pathway of peer learning was the presentations by “experienced” participants, showing concrete examples about a variety of research designs, their usage (match between CQ/RQ and designs), or research processes. The direct pathway was the questions asked by experienced participants to beginners, which were specific and concrete based on their recent research experience and helped beginners refine their research plans. Both pathways seemingly covered essential learning contents, such as research questions, designs, or research processes, which are difficult to learn by self-study or beyond one's specialty.

It can also be noted that there were gaps in background knowledge among participants and that some beginners were frustrated by the timeline to required outcomes. Therefore, we recommend that beginners first learn about a variety of research designs before starting peer learning and that they are given concrete and detailed examples of the research process leading to the outcomes.

### 4.2. Clinical Research Education

In recent decades, the importance of clinical research literacy and skills has increased dramatically as evidence-based medicine has become central to clinical medicine. Concurrently, clinical research education is a significant challenge at NCCHD with many early-career physicians and physician-scientists. Reports on clinical research education have been increasing, including education for medical students and nurses. Papers on clinical research education for physicians have been compiled from the USA, reporting a curriculum combining core lectures, seminars, small group learning, protocol development and research practice, and individual teaching and workshops on research tools [8–11]. However, there have been very few reports on the effects of peer learning in clinical research education, which were explored in this study.

### 4.3. Peer Learning

In peer learning, i.e., learning and education among peers, the usefulness of an “intermediary” who is positioned between beginners and experts and who plays both the role of learning and teaching has been pointed out [4]. That intermediary is an existence that evidently corresponds to the “experienced” category in this study. Some literature makes a strict distinction between peer learning, i.e., teaching between peers, and near-peer learning, i.e., teaching by slightly older or more advanced students [5]. From this perspective, the present study might be more of the latter type as described earlier, because it focused on learning between experienced participants and beginners. In any case, peer learning in a broad sense is not a simple model in which the teacher teaches the student, but rather a learning model in which the student both teaches and is taught in some situations. In that model, Vygotsky's theory of learning in the “zone of proximal development (ZPD)” is applied [5, 25].

ZPD, which provides the theoretical foundation for peer learning, refers to the level that cannot be reached by oneself but can be reached with some assistance [25]. ZPD is considered to be the area where peer learning is particularly useful [5]. We believe that the current peer-learning effects for content difficult for self-study, as abstracted in Figure 3, exactly tapped into the ZPD of beginners in clinical research.

### 4.4. Activity Theory

Engestrӧm's “activity theory” [16], which we used as the analytic framework for this study, is theoretically based on Vygotsky's works. Activity theory provides a model of group learning [16–24] that is an extension of Vygotsky's individual learning. In fact, both the whole model and the concepts of the theory, such as “instrument,” “outcome,” “community,” or “division of labor,” were valuable for our analysis.

There were some concepts of activity theory framework from which narratives could not be extracted in the interviews: “subject,” “object,” and “rule.” We believe that the failure to elicit those narratives is not fatal to this pilot study, and there may be some practical suggestions from the theoretical framework and concepts instead. Regarding beginners as “subject,” our initial concerns included the burden on beginners when we set our research question as noted earlier and the participation requirements when we recruited, but we could not obtain these narratives. Second, CQ/RQ as an “object” was not directly discussed, but there were mentions of CQ/RQ examples accompanying those of research design as “instrument.” As a suggestion for practice, it may be possible to reexamine CQ/RQ when encountering the difficulty of matching research design, in addition to using research experience as narrated in interviews. Next, although there was no direct mention of “rule,” there may be some practical suggestions, such as a rule that the facilitator encourages experienced participants to speak during the moment of silence in the seminar that was narrated in the interviews. Finally, regarding “division of labor,” the narrative of roles of experienced participants and staff could be drawn out, but not the role of beginners. One of the possible practical suggestions would be to reconsider beginners' labor if their burdens are too heavy.

Moreover, activity theory covers not only the learning effects on the individual but also the effects on the group or community as “expansive learning” [34]. Looking back from the “expansive learning” perspective, clinical research in the modern era is inevitably carried out collectively rather than individually at all stages of research plan, implementation, data analysis, and discussion or interpretation. The research outcomes surely contribute to society rather than the individual. In this context, the effects of clinical research education should be considered not only on the individual but also on the group. In particular, highly specialized medical institutions, such as NCCHD, are expected to actively promote evidence-generating clinical research. Therefore, we believe that our future prospects for clinical research education should be conducted with an awareness of the collective effects on both early-career physicians and physician-scientists who aim to generate evidence.

### 4.5. Implication and Reflection

We believe that the implication of this study related to our research question is that the extracted effects and points of peer learning suggest the significance and considerations for continuing peer-learning trials in clinical research education. The credibility of this implication depends on the subjectivity of the researchers, as is the nature of qualitative research. In this respect, the fact that one researcher, who was not involved in the seminar, attended the analysis would enhance research triangulation and thus credibility. On the other hand, relatively short interviews might set the limits of credibility.

We believe that the summarization of this study has a certain degree of transferability to clinical research education for physicians regardless of medical departments, as narratives and effects were extracted beyond the participants' specialties. On the other hand, there may be limits to transferability beyond the scope of postgraduate education because several narratives were inextricably based on physicians' clinical experience, such as narratives about clinical questions closely tied to specific disease names, or narratives like “Does this research result change clinical practice?”

### 4.6. Strengths and Limitations

The strength of this study is that we were able to obtain credible data from young physicians participating in peer learning, using semistructured interviews while their memories were still fresh.

Limitations of the study include the limited number of departments of the physicians studied due to the nature of the institution and the fact that the interview data were extracted from only one course of the seminar, which may limit the extracted content of the data. On the positive side, we were able to elicit some content not limited to pediatrics and perinatal medicine. Relatively short interviews with focused questions may not have reached theoretical saturation and elicited narratives on all the concepts of the theoretical framework, although for this study, saturation was not originally intended or essential, and the failure to elicit some narratives is not fatal as discussed above. In addition, combined with our research question focused on the beginners' experience, we did not extract learning effects other than those from experienced participants to beginners, e.g., in the opposite direction or between experienced participants, as reported previously [1, 2, 6, 7].

Despite these limitations, we believe that there are some implications and suggestions for peer-learning trials as discussed above. Our future tasks are to deepen and broaden the scope of clinical research education, as we advance the education programs with these pilot results and conduct further research.

## 5. Conclusions

In a clinical research seminar for physicians in a children's and mothers' hospital, the following learning effects of peer learning were extracted: the indirect pathway of presentations by experienced participants and the direct pathway of questions from experienced participants to beginners. The notes to consider were also extracted as gaps in background knowledge and the frustration of beginners. It is necessary for us to further develop the clinical research education program with an awareness of the usefulness and the notes to consider regarding peer learning, as well as the collective learning effects.

## Figures and Tables

**Table 1:** Extracted topics and text examples in familiarization step.

Extracted topic	*Text example (in italics)*
(1) “remarks” and “advice” that “leave” an “impression”	*Are there any other presentations, remarks, questions, or advice that left a lasting impression on you?*†

(2) “showing” a “direction”	*I think they have been teaching me, or rather, showing me in the right direction.*

(3) “teaching” and “stimulating” people to “notice,” “see,” and “know” the “problems” or “process” leading to the “final” “goal”	*There are a lot of problems that I do completely not notice, even in front of me. They may not teach me directly, but they may stimulate me to notice. They are asking what kind of process is easier to reach the final goal.*

(4) “telling” and “saying” to “think” about “options” of “designs” for “clinical questions” in the “paper”	*They tell us that maybe design is the most difficult part of the research process, from clinical questions to PECO, thinking about what kind of design should be used. They say there are many options, and what you see depends on them, and the impact of the paper is different.*

(5) “ringing” a “bell” about “getting” “data” and “results” being “out”	*If you proceed with the research using that design, and if it works, you can get these results out and write one paper with these results, do you think that's great? They are more likely to ring a bell if the question is like that.*

(6) Other words that could not be connected as clumps: “entirely,” “seminar,” “first,” “pediatrics,” “feel,” “clinical research,” “really,” “place,” “submit,” and “protocol,” “around”	*Entirely, I did not have a clear idea of what I was aiming for when I first heard about the seminar.* *This seminar, at first, was much in the mode of stimulating beginners to become aware, and there were situations when they waited for someone to start talking.* *Some said that if they were pediatrics researchers, they might understand, but if they were from totally unrelated departments, they might not feel much significance.* *I've never done clinical research before, so it was very good to see a precedent of how this kind of process works. I had no idea, I really did not know anything.* *In fact, when I stand in that place, perhaps I will remember.* *My goal is to submit for a grant in the fall. I thought that it would be too late to complete the protocol around the end of all sessions.*

We extracted five topics in the cooccurrence network diagram (Figure 1) made by the KH coder. We compared the network diagram with the raw texts in this familiarization step. We also reviewed and carefully read the words that were difficult to capture as clumps in the network diagram in the context of the raw texts. The extracted topics are listed on the left and text examples (in italics) on the right. †This text example was an interviewer's question. In this familiarization step, we included not only the participants' answers but also the interviewer's questions in the analysis to fully understand the context.

**Table 2:** Extracted process from qualitative analysis.

Label by activity theory	Extracted process	*Text examples (in italics)*
(a) Instrument: research design	Awareness that difficult research designs can be learned when encountering the required objects (CQ/RQ)	*If I experienced the same kind of difficult design for my own research, I would probably be able to understand.* *I have tried various designs and thought that the final design was quite good and mastered it.*
	The importance of knowing that there is a wide variety of research designs	*If you have a clinical question, there are a number of things you can do.* *What you can and cannot say depends on design.* *As long as you know that there are different designs, you can consult the experts.*
	The importance and difficulty of match of design with CQ/RQ and the significance of experiences	*When there is a clinical question, the most difficult part is to decide what kind of research design to use.* *I had a hard time in this area, so I have the experience of overcoming this difficulty.*

(b) Outcome: research result	Reexamining the designs by looking backward from the expected results	*Since you are interested in this, would you be happy if you could get this kind of data from that design, for example?* *Does this result from this design change clinical practice?*
	Expression of frustration with the timeline of the required outcomes	*The goal is clear, and the deadline is imminent, so I feel it's necessary to move fast toward that goal.* *I think I must speed up the process.*

(c) Community: seminar	Moments of silence in the seminar due to the gap in the participants' background knowledge of the instruments (research design and its match with CQ/RQ)	*Eventually there was a moment of silence at the end. Maybe it's because something like a research framework is not formed for beginners and experienced participants have experience and a framework.* *Frankly, I did not feel that it rang a bell.*
	The significance and interest of presentations beyond one's specialty	*They're talking about areas that are completely unfamiliar to me, outside of my area of expertise, and I think it's fun to hear about other areas.* *I thought the presentation conveyed the importance of the research to non-specialist physicians.*

(d) Division of labor: roles of participants and staff	Different roles between staff and “experienced” participants	*I think “experienced” participants are going to encourage, or rather stimulate, or interact with beginners and get the discussion going. Finally, staff will put a sort of seal on it.* *Staff was talking about the bigger goals of the research, not just the small point in front of me.*
	The role of “experienced” participants: (1) presentation providing concrete examples of CQ/RQ, designs, and process to outcomes	*In their presentation, I was very glad to see the precedent of how “experienced” participants have done this kind of process and had these problems.* *I know the name of AMED or ERB, but have no idea what steps to take, so watching their presentation, I would say, “Oh, so that is the way to do it”.*
	The role of “experienced” participants: (2) specific and concrete questions based on recent experiences	*I think that was probably the point they were struggling with so much, so they asked us that question.* *The questions are probably questions that “experienced” participants themselves were asked last year and I think they are very important and helpful.*

This table shows the charting results of the qualitative analysis. In the analysis, interview texts were intercepted, decontextualized, and coded using the labels generated in Figure 2 based on the activity theory by Engestrӧm. Then, the texts were recontextualized and process-formed. Shown from left to right are labels, extracted processes, and text examples. CQ: clinical question; RQ: research question.

## Data Availability

The data used to support the findings of this study are available from the corresponding author upon request.

## Abbreviations

NCCHD: National Center for Child Health and Development
CQ: Clinical question
RQ: Research question
ZPD: Zone of proximal development.
